# Plasma Gelsolin: Indicator of Inflammation and Its Potential as a Diagnostic Tool and Therapeutic Target

**DOI:** 10.3390/ijms19092516

**Published:** 2018-08-25

**Authors:** Ewelina Piktel, Ilya Levental, Bonita Durnaś, Paul A. Janmey, Robert Bucki

**Affiliations:** 1 Department of Microbiological and Nanobiomedical Engineering, Medical University of Bialystok, Mickiewicza 2c, 15-222 Bialystok, Poland; ewelina.piktel@wp.pl; 2 McGovern Medical School, University of Texas Health Science Center, Houston MSB 4.202A, 6431 Fannin St, Houston, TX 77096, USA; Ilya.Levental@uth.tmc.edu; 3 Department of Microbiology and Immunology, The Faculty of Medicine and Health Sciences of the Jan Kochanowski University in Kielce, Aleja IX Wieków Kielc, 25-317 Kielce, Poland; Bonita.Durnas@onkol.kielce.pl; 4 Institute for Medicine and Engineering, University of Pennsylvania, 3340 Smith Walk, Philadelphia, PA 19104, USA; janmey@mail.med.upenn.edu

**Keywords:** plasma gelsolin, inflammation, biomarker, extracellular recombinant gelsolin, actin

## Abstract

Gelsolin, an actin-depolymerizing protein expressed both in extracellular fluids and in the cytoplasm of a majority of human cells, has been recently implicated in a variety of both physiological and pathological processes. Its extracellular isoform, called plasma gelsolin (pGSN), is present in blood, cerebrospinal fluid, milk, urine, and other extracellular fluids. This isoform has been recognized as a potential biomarker of inflammatory-associated medical conditions, allowing for the prediction of illness severity, recovery, efficacy of treatment, and clinical outcome. A compelling number of animal studies also demonstrate a broad spectrum of beneficial effects mediated by gelsolin, suggesting therapeutic utility for extracellular recombinant gelsolin. In the review, we summarize the current data related to the potential of pGSN as an inflammatory predictor and therapeutic target, discuss gelsolin-mediated mechanisms of action, and highlight recent progress in the clinical use of pGSN.

## 1. Introduction

Gelsolin (GSN) is a multifunctional protein with actin filament severing, capping, and nucleating activity, implicated mainly in the remodeling of cytoskeletal structure, which ultimately determines cell shape, chemotaxis, and secretion [1]. Factors regulating actin-binding properties of gelsolin include pH, phosphoinositides, lysophosphatidic acid, and Ca^2+^ [1]. The ~82–84 kDa protein consists of 730 amino acids organized into six homologous domains (G1 through G6), with each one implicated in one or more biological functions of GSN [2]. Homologous repeats of gelsolin-like domains have been found in other actin-remodeling proteins (e.g., flightless I, adseverin, capG, villin, advillin, and supervillin) that together with gelsolin form the gelsolin superfamily. A number of studies aiming to evaluate the structure and cellular functions of gelsolin-related proteins demonstrated that adseverin shares 60% amino acid homology with gelsolin (but lacks a C-terminal helix found in gelsolin), whereas flightless I, villin, advillin, and supervillin are characterized by additional domains beyond the six-fold repeat of the gelsolin-like domain. In contrast, CapG only contains three gelsolin-related domains, which limits the actin severing activity of this protein [3]. Among a variety of cellular functions noted for gelsolin-related peptides, regulation of actin dynamics, impact on cell motility, phagocytosis, apoptosis, platelet formation, and exocytosis are the most prominent [3].

An important distinguishing feature of GSN among other mammalian cytosolic proteins is the presence of a secretory isoform: plasma gelsolin (pGSN). Both of these forms, cytosolic and extracellular, are coded by the same gene on the ninth chromosome and exert the ability to depolymerize actin filaments; however, they differ by an additional 24 residue sequence, termed the “plasma extension”, and the presence of a disulfide bond between Cys^188^ and Cys^201^ in the G2 domain of pGSN, which enhances the stability of the secretory isoform in the extracellular environment [4,5]. Some studies also support a third minor isoform of GSN, called gelsolin-3, expressed primarily in oligodendrocytes in the brain, lungs, and testes, and involved in myelin remodeling during wrapping of the axon [6]. The presence of extracellular forms was reported also for other members of the gelsolin superfamily, primarily flightless I, secreted by cells found in wounds (fibroblasts and macrophages) as well as extracellular matrix, plasma, acute and chronic wound fluid, and blister wound fluid, where it negatively regulates wound and burn injury repair [7]. 

### Multifunctional Role of Gelsolin in Health and Disease

Extracellular gelsolin circulates in mammalian blood at concentrations of 150–300 μg/ml, with some variations depending on the detection method employed. Serum levels of gelsolin are reported to be lower than plasma levels due to an interaction between GSN and fibronectin and/or fibrin [8]. In contrast to cytoplasmic gelsolin (cGSN), which is ubiquitously expressed, expression of pGSN is more limited. Currently, skeletal, cardiac, and smooth muscles are recognized as the main sources of plasma gelsolin in the bloodstream [9], although relatively high amounts of this protein are secreted by cells of the central nervous system (CNS) and are also detected in airway surface fluid (ASF) [10] resulting from secretion from IL-4-activated lung epithelial cells. Gelsolin has also been detected in lymph [11], burn wound fluid [12] and cerebrospinal fluid (CSF), though GSN concentration in CSF is approximately twenty times lower than in plasma [13,14]. Recently, gelsolin was also reported to be present in mid-trimester amniotic fluid [15].

**Figure 1:**
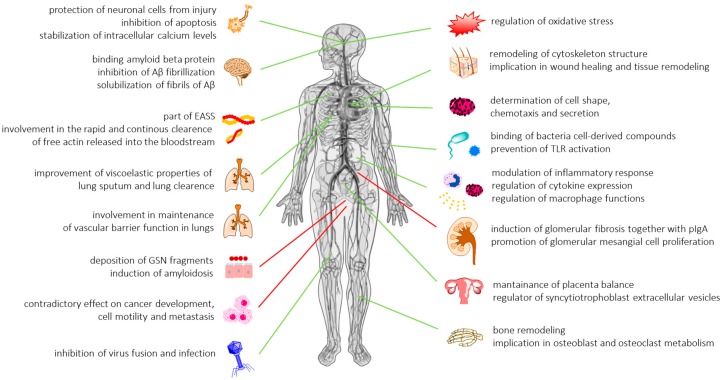
The implications of extracellular gelsolin in a variety of physiological and pathological processes. Green arrows indicate the beneficial effect of gelsolin in the maintenance of health and physiological balance; red arrows indicate disadvantageous gelsolin-mediated events.

Available data strongly support a crucial role for gelsolin in a variety of physiological and pathological processes (Figure 1), which have been thoroughly discussed previously [2,16]. To date, a majority of gelsolin-aimed research has been focused on GSN-mediated remodeling of the cytoskeleton and the role of pGSN as a part of the extracellular actin scavenger system (EASS) responsible for rapid and continuous severing and removal of actin filaments released from dead cells into the bloodstream [17]. Actin is present in high concentrations in most eukaryotic cells, constituting more than 10% of the cellular protein [18,19]. The release of actin into systemic circulation in response to injury or illness-associated necrosis results in adverse pathophysiologic consequences including (i) increase of blood viscosity and disturbances in microvascular flow, (ii) activation of platelets with resulting platelet aggregation, (iii) microvascular thrombosis, (iv) release of proinflammatory mediators such as thromboxane, (v) impairment of fibrinolysis, (vi) promotion of alpha-haemolysin (HlyA) production, exacerbating *Escherichia coli* infections, and (vii) binding of adenine nucleotides that could activate purigenic receptors [19,20,21,22,23,24]. Thus, actin exposure resulting from primary injury can lead to the development of secondary tissue injury due to the high toxicity of actin. Consistently, a significant decline of pGSN in patients in a variety of injury states and major/minor trauma is observed and often predicts complications of injuries such as changes in lung permeability and death.

**Figure 2:**
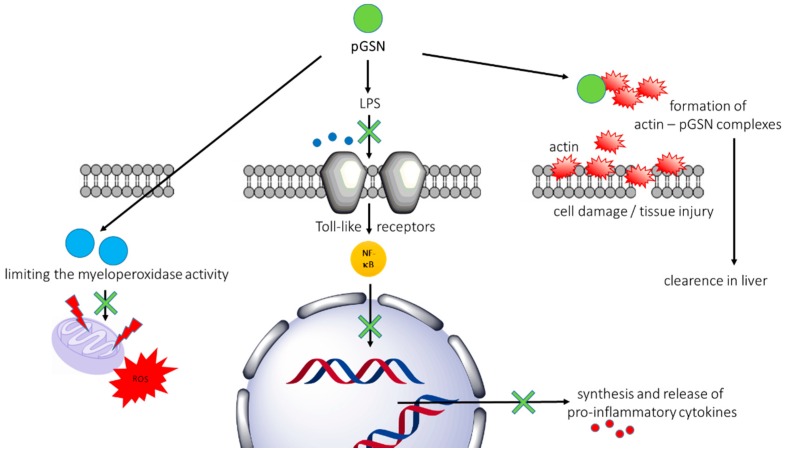
The protective role of gelsolin against oxidative stress, microbial-induced inflammatory states, and toxic effects of actin released from damaged cells and tissues.

In contrast to the well-known actin scavenging and clearance activity of gelsolin, there are a limited number of data about other functions of gelsolin’s isoforms present in extracellular fluids. For this reason, studies focused on pGSN were extended to evaluate the mechanisms by which gelsolin is involved in apoptosis, signal transduction, epigenetic processes, transcriptional regulation, modulation of inflammatory response, and pathogenesis of diseases. Consequently, apart from its role in sequestering actin in extracellular compartments, it was established that pGSN modulates immune responses by preferential binding of bacterial cell wall-derived compounds, i.e., lipopolysaccharides (LPS) and lipoteichoic acid (LTA) from Gram-negative and Gram-positive bacteria, respectively. Gelsolin can also prevent toll-like receptor (TLR) activation, due to the structural similarity between intracellular gelsolin-binding molecules (phosphatidylinositol 4,5-bisphosphate and lysophosphatidic acid (PIP2 and LPA)) and bacterial endotoxins [25]. In addition, pGSN binds to a broad spectrum of bioactive compounds, including LPA, sphingosine-1-phosphate (S1P), and platelet activating factor (PAF), enhancing the protective properties of GSN in inflammatory states, and suggesting a role in processes such as wound healing, tissue remodeling, cancer development, or angiogenesis (Figure 2) [26,27,28]. These cellular effects partially overlap with the roles of other gelsolin superfamily members. Previous studies demonstrated that in addition to their impact on actin filament remodeling, gelsolin-related proteins (incl. flightless I, capG and adseverin) play roles in diminishing LPS- and TLR-mediated inflammation processes, wound healing, osteoclastogenesis, and endothelial cell response to mechanical forces [29,30,31]. These results highlight the diversity of biological roles of gelsolin, and due to its strong impact on a broad spectrum of cytoskeleton- and inflammatory-related biological processes, its potential as biological indicator in a variety of diseases and medical conditions.

An important step in revealing the critical biological functions of gelsolin was the generation of transgenic gelsolin-lacking (gsn −/−) mouse models. Witke et al. demonstrated that despite normal viability and fertility, gelsolin-null mice exhibited a number of defects in actin-mediated processes, including decreased platelet shape changes and activation leading to prolonged bleeding times after injury. Also observed were dysfunctions in inflammatory reactions, namely delayed in vivo migration of neutrophils into peritoneal exudates. Further disturbances were excessive actin stress fibers, impaired chemotaxis, and increased contractility in gelsolin-lacking dermal fibroblasts, highlighting a role of gelsolin in responses requiring fast motility under stress conditions [32]. Gelsolin was also found to be essential for proper wound healing, since gelsolin-knockout fibroblasts reveal a significant defect in healing processes [32]. Gelsolin-deficient mice were also characterized by increased susceptibility of hippocampal neurons to glutamate-induced excitoxicity and exacerbations of seizure-induced damage to hippocampal pyramidal neurons, highlighting a neuroprotective role of gelsolin [33]. Research performed using mouse models of pain and acute inflammation suggested analgesic and anti-inflammatory properties of GSN [34]. Further studies using gelsolin-null mice revealed a role for gelsolin in cancer development, pathogenesis of diabetes, neurodegenerative diseases, and involvement in osteoblast metabolism [35,36,37]. In order to accurately evaluate the role of plasma gelsolin in these processes, it is crucial to develop animal models characterized by the knockout of extracellular gelsolin only, not of total GSN. To date, such optimal models have not been introduced, which emphasizes the need for caution in assessing the role of pGSN in these observations.

In agreement with the above-mentioned reports, an increasing number of studies reveal that GSN blood levels are altered in some critical medical conditions, including trauma, septic shock and multiple organ dysfunction syndrome (MODS), neurodegenerative diseases, cancers, chronic inflammatory disorders (e.g., rheumatoid arthritis, chronic kidney diseases, and multiple sclerosis), and hormone-associated illnesses (e.g., diabetes). These observations suggest a complex role of extracellular gelsolin in the pathogenesis of these conditions and its potential as a diagnostic marker, particularly as a part of multifactor diagnostic panels.

## 2. Alterations in Plasma Gelsolin Concentrations in Different Clinical Conditions

### 2.1. Trauma

#### 2.1.1. Release of Actin from Damaged Tissues as a Major Pathological Event in Trauma Patients

Systematic studies over the last few decades have shown that blood gelsolin levels are significantly decreased in humans and mice in a variety of conditions requiring urgent medical attention. These include tissue injury, development of secondary organ damage, and critical care complications such as burn-induced pulmonary microvascular dysfunction [38], acute oxidant lung injury [39], traumatic brain injury [40], MODS [41], radiation-induced injury [42,43], acute liver failure, myocardial infarction, septic shock, and myonecrosis [44] (Table 1). A crucial event in the pathogenesis of the majority of injury/trauma-induced conditions is the release of actin from damaged tissues into the systemic circulation, exceeding the protective function of actin scavenging systems (including pGSN). This accumulation of free actin, generally bound to adenosine diphosphate (ADP), induces an inflammatory reaction, which can contribute to severe organ damage. Free actin present in serum was also found to be toxic in vitro using cultured sheep pulmonary artery endothelial cells [45]. Importantly, the degree of gelsolin decline in these patients is inversely correlated with the duration of mechanical ventilation, the duration of intensive care unit (ICU) stay, overall hospital stay, illness severity, and overall in-hospital mortality [18,44].

#### 2.1.2. Decreased Concentrations of pGSN in Trauma Patients

According to Mounzer et al., low plasma gelsolin levels (> 2 standard deviations below the mean of the control group) appear to be an early predictive marker in patients experiencing major trauma, distinguishing patients that developed acute respiratory distress syndrome (ARDS) from subjects with lesser injuries and better survival predictions [18]. Low plasma gelsolin levels might also predict clinical outcomes in patients with severe burns and major surgical procedures. In contrast, a correlation between pGSN concentrations and ARDS development was not noted in a different study, presumably due to the inclusion of patients with more severe conditions [46]. In burn patients, plasma gelsolin levels decreased with increasing burn sizes, and increasing incidence of septic complication, and correlated with development of multiple organ dysfunction syndrome and enhanced burn- and sepsis-associated mortality [47,48]. These complications are likely determined by accumulation of free actin in burn-wound fluid and gelsolin inactivation at the wound site due to proteolysis or inactivation [12]. Importantly, for proper employment of pGSN concentrations as a useful predictor for disease severity and prognosis of recovery, daily measurements of plasma protein are required, since levels of GSN varied in different postburn days (PBD), with the lowest gelsolin level observed 7 days after the burn, not on the admission day or the first 3 days, as might have been expected for an acute injury. Gradual recovery of gelsolin concentration is most likely associated with effective treatment, which would be in line with a study demonstrating greater than two-fold increases of gelsolin blood levels resulting from continuous sedation therapy in patients with severe burn-induced sepsis [47,48,49]. The predictive value of lowered gelsolin concentration, similar to that of Glasgow Coma Scale (GCS) score, was also noted in patients with traumatic brain injury (TBI) [50]. An interesting study focused on the predictive effectiveness of plasma gelsolin in patients below 3 years of age developing cardiopulmonary bypass-induced acute lung injury (CPB-ALI) as the result of cardiac surgery. This study demonstrated that not only blood concentration, but also the kinetics of protein decline, may be early predictors of clinical outcome. According to Shi et al., infants and young children undergoing cardiac surgery developing CPB-ALI not only have a lower pGSN preoperative reservoir, which makes them more prone to acute lung injury after CPB, but their plasma gelsolin was consumed much earlier after the operation when compared to non-ALI patients. It was suggested that the more rapid decline of pGSN between the ALI and the non-ALI group (6 h after CPB vs 48 h) is associated with the clearance of circulating actin released from damaged tissues and the binding of inflammatory mediators and pro-inflammatory cytokines, the release of which is initially provoked by CPB [51].

Plasma gelsolin levels also drop shortly after hematopoietic stem cell transplantation, and bone marrow transplant patients are more likely to develop respiratory complications, including idiopathic pneumonia syndrome (IPS) [52]. Similarly, in patients with acute liver injury, low levels of pGSN correlate with serum aminotransferase and bilirubin levels and its fall depends exclusively on the extent of actin leakage from the injured liver, since plasma gelsolin is not a hepatic protein [53]. These data strongly suggest that plasma gelsolin depletion might be a sensitive predictor of secondary inflammation and tissue injury.

#### 2.1.3. Repletion of Physiological Level of pGSN as a Therapeutic Option in Trauma Subjects

Owing to the protective role of extracellular gelsolin against injury-induced actin toxicity and inflammatory response, the use of gelsolin to restore physiological (or supraphysiological) levels of pGSN has been suggested as a potential therapy in a variety of lethal conditions (Table 2). Previously, plasma gelsolin has been isolated from human plasma; however, this method is characterized by a broad spectrum of limitations including lack of purity, risk of blood-borne diseases, and low process efficiency. Currently, gelsolin is produced using recombinant hosts, such as mammalian cells or *E. coli*. Although pGSN produced in *E. coli* lacks the disulfide bonds of native pGSN, it can be converted to the plasma-like conformation using mild oxidation [5]. In effect, the *E. coli*-derived product is characterized by the same folding and presence of disulfide bonds as native human gelsolin, which enables its use for pGSN-based therapeutic approaches [4]. To date, a few (mainly mouse model-based) studies focused on evaluating the therapeutic efficiency of gelsolin repletion in subjects with insult-induced acute lung injury. In accordance with data demonstrating the involvement of gelsolin in maintenance of lung physiology [39,45,90,91,92], Christofidou-Solomidou et al. [39] and Rothenbach et al. [38] demonstrated that exogenous GSN might serve a protective role against lung injury. In a model of hyperoxia-induced lung injury, administration of recombinant human gelsolin can diminish the characteristic acute inflammatory response [39]. The beneficial effect of exogenous gelsolin was determined primarily by the limitation of neutrophil migration, scavenging of soluble pro-inflammatory mediators, and inhibition of neutrophil adhesion to endothelial surfaces [39]. This study suggested that plasma gelsolin levels at least 25% of normal could be a critical level for maintaining normal physiology, since mice suffering from hyperoxia-induced lung injury were found to have a plasma gelsolin level below 25% of normal. Similarly, in bone marrow transplant patients, the development of IPS, fatal respiratory complications, and increased mortality was preceded by hypogelsolinemia of ~25% of normal blood value [52]. Considering these results, the repletion of human gelsolin level above this critical point by exogenous administration of GSN may be considered to be beneficial in prevention of injury-induced complications. Consistently, intravenous infusion of recombinant gelsolin prevents inflammation-induced pulmonary microvascular dysfunction in burn-injured rats by ameliorating the toxic effects of actin released into the microcirculation and altering the bioactivity of LPA [38]. The outcomes of mice following major burns are also improved by recombinant gelsolin by reducing neuroinflammation and apoptosis in the brain, and by improving peripheral T lymphocyte function [84]. In addition to these reports, Li et al. suggested the ability of pGSN to block radiation-induced injury, which was supported by previous studies demonstrating the antioxidant effect of GSN [39], which is mediated through lipid peroxidation suppression, an increase in antioxidant generation, and shortening of bleeding time [42]. The promising results of research using mouse models subjected to a variety of acute insults indicate that administration of plasma gelsolin to patients with low pGSN levels and at risk for critical care conditions is worthy of consideration.

### 2.2. Sepsis

#### 2.2.1. Prognostic Value of pGSN in Sepsis Patients When Combined with Other Sepsis Biomarkers

Epidemiological studies suggest an increasing incidence of sepsis from microbial infections triggered mainly by the release of LPS/LTA molecules from the outer wall of Gram-negative or Gram-positive bacteria. Due to the difficult pathophysiology of this syndrome (complex chain of events including inflammatory and anti-inflammatory processes, humoral and cellular reactions, circulatory abnormalities, and the highly variable and non-specific nature of symptoms), severe sepsis and septic shock remain a leading cause of death at the intensive care units (ICUs) with a mortality rate up to 72.1%. This fact motivates the search for new disease markers, allowing for early diagnosis and evaluation of sepsis severity [93,94]. To date, more than 170 different biomarkers have been evaluated for use in sepsis complications and outcome prediction, evaluation of therapy response, and development of organ dysfunction [95]. Despite this number, only a few of them, including C-reactive protein (CRP) [96] and procalcitonin (PCT) [97,98], are routinely employed in clinical practice due to low specificity or sensitivity of the proposed biomarkers [95,99]. Even CRP and PCT therapeutic values have been challenged due to difficulties in distinguishing sepsis from other inflammatory diseases [98,100,101].

The potential usefulness of pGSN as a sepsis biomarker is encouraged by reports indicating depletion of plasma gelsolin both in an animal model of sepsis and in critically ill surgical patients, particularly those diagnosed with severe sepsis [17,44,102]. Suhler et al. reported a significant reduction in mean gelsolin concentrations when compared with healthy individuals in 51% of patients with sepsis, which correlated with illness severity [44]. Further studies confirmed that admission plasma gelsolin levels are also lower in neonatal infants and adult patients with severe sepsis than in non-septic critically ill patients in the ICU, including those diagnosed with systemic inflammatory response syndrome (SIRS) [17,54,102]. Simultaneous measurement of pGSN depletion with other well-known biomarkers might increase the diagnostic sensitivity and specificity of extracellular gelsolin-based assessments. Despite the correlation between occurrence of severe sepsis and admission pGSN level, Wang et al. did not observe statistically significant differences in gelsolin concentration between surviving and non-surviving patients with severe sepsis [17]. These results suggest that depletion of pGSN alone might be useful in differentiation between the septic and non-septic groups and could facilitate the early diagnosis of sepsis, but does not provide clear information about the sepsis outcome prognosis. This result opposes the report by Horváth-Szalai et al. indicating higher GSN levels in sera of survivors than non-survivors [102]. Simultaneously, it was confirmed the plasma gelsolin level recovery was correlated with clinical improvements in survivors of severe sepsis, which clearly indicates that daily assessments of pGSN concentrations are required for accurate sepsis case prediction [17,103]. Based on these reports, it was proposed that apart from serum GSN concentrations measurements, simultaneous assessment of serum actin and determination of actin/gelsolin ratio (A/GSN ratio) might be useful in the evaluation of SIRS and sepsis severity. Horváth-Szalai et al. demonstrated that A/GSN ratios were not only significantly higher in sera of septic patients than in control subjects, but also the second day’s A/GSN ratios were significantly higher in non-survivors than survivors. A/GSN ratios positively correlated with CRP levels and sequential organ failure assessment (SOFA) clinical scores, supporting its potential as a novel prognostic marker for sepsis. Nevertheless, its clinical applicability is limited by the lack of rapid laboratory methods for determination of actin and gelsolin in patients’ samples [102]. In response to this issue, novel turbidimetric measurements of gelsolin have been developed, offering a rapid and accurate quantitation of gelsolin in human serum samples; nevertheless, more accurate analyses are needed to introduce this analytic method into clinical practice [104]. Another proposition from this research team involves the simultaneous determination of plasma gelsolin and Gc-globulin. According to a recent study, both serum GSN and Gc-globulin have potential as predictors of sepsis severity and sepsis-associated mortality. This study also confirmed that the pGSN concentration was higher in sepsis survivors than in non-survivors [105].

#### 2.2.2. Beneficial Gelsolin-Mediated Mechanisms in Animal Models of Sepsis

Apart from the actin scavenging properties of plasma gelsolin, one of the most important features of extracellular gelsolin for its protective role in sepsis is the ability of pGSN to selectively interact with bacterial wall products (i.e., LPS from gram-negative, and LTA from gram-positive bacteria), effectively modulating cellular response and diminishing the inflammatory reaction of the host. To date, a few studies demonstrated that due to structural similarity between bacterial endotoxins and PIP2/LPA, recombinant pGSN and/or a rhodamine-B-conjugated peptide synthetized based on gelsolin’s PIP2-binding sequence (PBP10) (i) bind LPS from various bacteria, including *Escherichia coli*, *Salmonella enteritidis*, *Pseudomonas aeruginosa*, or *Klebsiella pneumoniae*, (ii) interact with purified LPS and LTA, (iii) exert antibacterial activity against both gram-positive and gram-negative bacteria due to interaction of PBP10 with the negatively charged bacterial wall lipids, and (iv) prevent endotoxin-mediated toll-like receptor activation [25,106,107,108,109]. Administration of pGSN to animals subjected to systemic inflammation can prolong survival, prevent complications of acute injury [38,83], and inhibit LPS-induced inflammatory process both in vitro and in vivo [110]. These findings suggest that pGSN is a broad-spectrum anti-inflammatory buffer and that local pGSN depletion allows mediators to promote inflammatory response of the host. In the case of prolonged pGSN depletion, the dysfunctional and destructive actions of the mediators may lead to secondary organ damage and even death.

The above reports clearly indicate that plasma gelsolin might be beneficial in therapy of sepsis. Using a mouse model of sepsis, Lee et al. note the decrease of pGSN levels to ~50% of control adult mice 6 h after subjection of animals to lethal doses of LPS or polymicrobial challenge after cecal ligation and puncture (CLP). pGSN remained low for ≥ 24 h after the insults, which was presumably determined by the release of actin as part of cellular injury, resulting in actin filament formation and further tissue injury and organ dysfunction [85,107,111]. Importantly, it was noted that subcutaneous or intravenous injection of exogenous pGSN after LPS challenge or CLP in a dose ~20 times higher than total gelsolin concentration in blood (calculated based on the weight of the animal and volume of blood), significantly improved illness outcome via solubilization of circulating actin aggregates in both mice and rat model of sepsis [85,86]. Repletion with exogenous gelsolin improved survival rate of both endotoxemic and CLP-treated mice and rats, and altered inflammatory cytokine expression 24 h after insult [85,86], which emphasizes the validity of reversing pGSN deficiency as an effective treatment option for sepsis. The putative mode of action was modification of systemic inflammatory response via binding and neutralizing bioactive inflammatory mediators, as well as the actin filament-severing and sequestering properties of pGSN [85]. This inference was supported by results showing that CLP-initiated illness was ameliorated by injection of pGSN either subcutaneously or intravenously, but not intraperitoneally, pointing out that pGSN must act systemically, not exclusively at the site of injury, in order to be effective as an anti-sepsis factor [86]. Thus, not only repletion to physiological pGSN level should be considered, but also potential benefits of artificially induced hypergelsolinemia.

### 2.3. Infections and Infection-Associated Diseases

#### 2.3.1. Advantageous Effect of Gelsolin on Host Anti-Infection Protection Mechanisms

Although direct effects of gelsolin on bacterial growth have not been observed so far [2], a few studies suggest the beneficial role of GSN in modulation of the host antimicrobial response, thus providing the possibility that gelsolin may be a sensitive infection biomarker and therapeutic tool. Gelsolin is known to play a role in Fc-receptor and integrin-mediated phagocytosis, but not in complement-mediated phagocytosis [112,113], and gelsolin-null mouse fibroblasts are characterized by defective Fcg-receptor-mediated phagocytosis [112]. Previously, it was demonstrated that pGSN protects animals from microbial-induced septic shock resulting from peritoneal administration of bacterial suspensions [85,86] and improves host defense mechanisms due to its impact on nitric oxide (NO) synthase type III (NOS3). In a mouse model of primary pneumococcal pneumonia, administration of recombinant human pGSN improved lung macrophage uptake and killing of bacteria (*Streptococcus pneumoniae*, *Escherichia coli*, and *Francisella tularensis*) due to phosphorylation of NOS3, an enzyme with important bactericidal functions in lung macrophages [87]. Plasma gelsolin was also noted to restore bacterial binding and uptake by murine and human macrophages, impaired by scavenger receptor MARCO-mediated interaction of free, released actin with macrophages [114]. In light of these reports, we evaluate here the predictive and therapeutic potential of plasma gelsolin in infections and infection-associated diseases.

#### 2.3.2. Varied Concentrations of pGSN in Bacterial, Protozoa and Viral-Induced Diseases

In 1988, pGSN concentrations were measured in patients with acute *Plasmodium falciparum* malaria infections, whose gelsolin levels were decreased to <50% of healthy controls, and whose levels increased after treatment with chloroquine. Animal studies, including acutely and subcutaneously induced hemolysis in rabbits, suggested that decline of pGSN was related to clearing the actin released from destroyed erythrocytes [56]. This hypothesis was confirmed by later research employing proteomic analysis, where the concentration of plasma gelsolin was noted to be significantly lowered in patients diagnosed with malaria due to (i) release of actin into peripheral blood and (ii) formation of complexes with hemozoin (i.e., a dark-brown crystal formed by the parasite and released into the host during the bursting of infected red blood cells) [57]. Argun et al. presented a correlation between pGSN levels and acute rheumatic carditis, a heart disease resulting from acute rheumatic fever caused by *Streptococcus* from group A and demonstrated the association of decreased plasma gelsolin levels with clinical parameters, including mitral regurgitation or left ventricular end-diastolic diameter [115]. One report suggested potential utility of increased plasma levels of gelsolin in diagnosing disease severity and evaluating efficacy of therapeutic treatments during *Trypanosoma cruzi* infection and Chagas disease [116]. A compelling study using a mouse model of *Streptococcus pneumoniae*-induced pneumonia demonstrated that even delayed administration of recombinant human GSN as single doses on days 2 and 3 after infection without antibiotics could substantially improve animal survival [117].

An interesting study proposed plasma gelsolin as a biomarker for hepatitis B-associated liver cirrhosis [58]. It is estimated that each year, ~1 million persons die from chronic complications resulting from infection with hepatitis B virus (HBV). Such complications are primarily liver cirrhosis, liver cancer, and liver failure, leading to progressive loss of liver function. In the absence of robust biomarkers for evaluation of hepatic fibrosis stages and progression of chronic HBV-associated diseases, as well as a number of limitations inherent to liver biopsy, the utility of plasma-based tests and serum markers, such as gelsolin, are particularly important. To date, proteomic analyses comparing the plasma proteome of inactive HBV-infected patients and patients suffering from HBV-associated liver cirrhosis confirmed repression of gelsolin in cirrhotic plasma samples. Further investigations are needed to confirm the utility of gelsolin as a prognostic marker or a therapeutic agent in liver cirrhosis [58].

**Figure 3:**
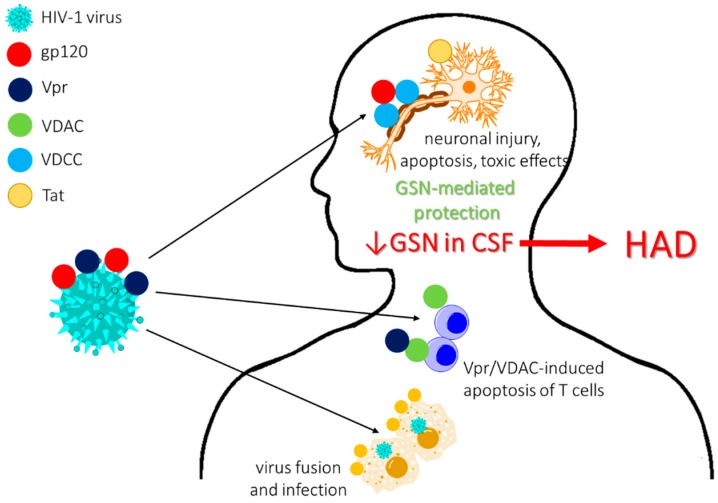
The neuroprotective and anti-infectious role of pGSN in development of HIV-1-associated dementia.

Significant data also suggest a protective role of gelsolin against HIV-1 infections due to (i) weakening HIV-1 Env-gp120-mediated F-actin reorganization and viral receptor capping required for virus fusion and infection and (ii) inhibition of T-cell apoptosis induced by HIV-Viral protein R (Vpr) via blocking the interaction between Vpr and voltage-dependent anion channel (VDAC) [118,119]. Liu et al. reported a neuroprotective role of pGSN as an agent suppressing gp120-induced neuronal injury by preventing the modification of voltage-gated K+ channel Kv2.1 expression [120]. Simultaneously, several reports of alterations in gelsolin concentrations in HIV-infected patients have been presented. Proteomic studies of CSF of patients diagnosed with HIV-1 associated dementia (HAD), a cognitive disturbance resulting from a metabolic encephalopathy caused by secretory products released from immune-competent and virus-infected brain mononuclear phagocytes, have revealed decreased pGSN levels in HIV-infected individuals [59], in agreement with reports of a protective role for pGSN in neuronal cell apoptosis (Figure 3) [120]. New research investigating pGSN expression in the cortex of the brains of rhesus monkeys infected with SIV (simian immunodeficiency virus) presented interesting conclusions: (1) neurons of virus-infected subjects do not secrete high amounts of pGSN; (2) pGSN protects neurons from toxic effects of the HIV protein Tat, which is in part mediated by blocking Tat–integrin interactions; and (3) pGSN accumulates in macrophage nodules formed in response to SIV infection of monkey brains creating locally high levels of this protein in the brain. Despite the accumulation of pGSN in macrophage nodules formed in response to SIV infection of monkey brains, this effect did not reflect the overall decreased level in the CSF compartment [14]. The other important observation from the same cohort of HAD patients was increased expression of gelsolin in HAD sera, which was undetected previously using a different analytical method and upregulated gelsolin levels in HIV-1-infected and HIV-/HCV-coinfected patients, induced likely by increased viral infection, which highlights the importance of the analytical method in evaluation of potential biomarkers [60,121].

#### 2.3.3. The Clinical Potential of Application of Plasma Gelsolin as Mucolytic Agent in CF Patients

**Figure 4:**
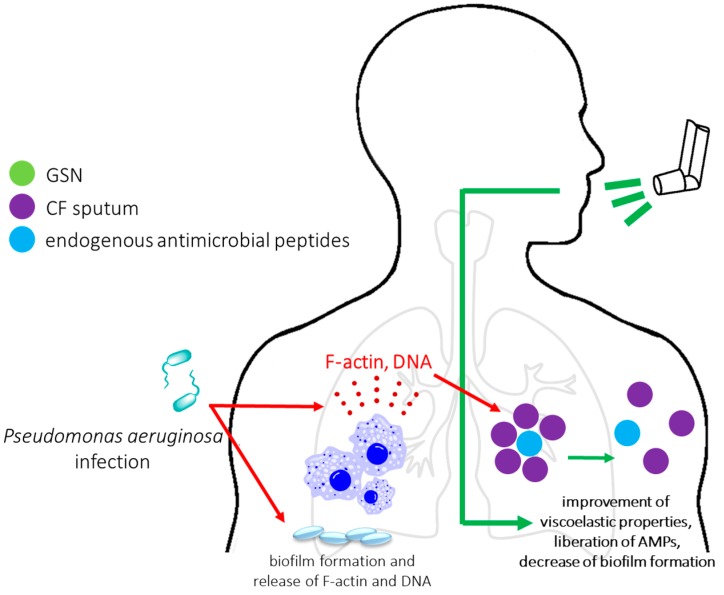
The mechanisms of therapeutic action of extracellular gelsolin administrated in the form of aerosol in cystic fibrosis patients infected with *Pseudomonas aeruginosa*.

Despite the promising results showing the significant prognostic and therapeutic potential of gelsolin, the most advanced clinical research has focused on the application of plasma gelsolin as a mucolytic agent aimed to decrease the abnormal viscoelasticity of cystic fibrosis (CF) sputum. A change in the viscoelasticity of sputum in CF is caused by accumulation of neutrophil-released DNA and F-actin, and is associated with dysfunction of ciliary transport, resulting in bronchial obstruction and chronic bacterial infections (mainly *Pseudomonas aeruginosa*) [122,123]. Endogenous antimicrobial agents (including human cathelicidin LL-37, beta-defensins, and lysozymes) are trapped in the CF sputum network, considerably limiting their antibacterial properties [124]. Therefore, it was proposed that disassembly of DNA/F-actin bundles with depolymerizing and actin severing agents, including pGSN, could fluidize CF sputum, improve its viscoelastic properties, increase lung clearance, and liberate antimicrobial peptides, leading to restoration of innate antimicrobial defense [124,125]. These observations justified the introduction of aerosolized gelsolin in phase II clinical trials, which demonstrated good tolerance in humans. Due to no significant improvement of lung expiratory functions (the only measured parameter), further clinical trials were suspended. Despite this, other valuable gelsolin-mediated features, including binding of bacterial endotoxins, prevention of inactivation of antimicrobial peptides [108], and reversal of the stimulatory effect of actin on *P. aeruginosa* biofilm production in CF lungs [126], indicate that further clinical studies on use of exogenous gelsolin in the treatment of chronic infections that occur in cystic fibrosis airways, alone or in combination with other depolymerizing agents (e.g., Pulmozyme; inhaled recombinant human deoxyribonuclease 1), are justified (Figure 4) [125].

### 2.4. Chronic Inflammatory and Autoimmune Disorders

#### 2.4.1. Limiting the Use of Gelsolin as a Disease Biomarker in Chronic Inflammatory Conditions

Chronic inflammatory systemic diseases (CIDs) like rheumatoid arthritis, systemic lupus erythematosus, multiple sclerosis, and many others lead to long-term suffering, stress, and high costs of treatment. Both chronic autoimmune and inflammatory diseases arise through abnormal reactions of the human adaptive or innate immune systems [127]. The protective properties of gelsolin as a broad-spectrum anti-inflammatory buffer and actin binding factor promote the hypothesis that depletion of pGSN in chronic inflammatory conditions leads to cellular damage and mediator release. Such actin- and inflammatory mediator-mediated diminution of pGSN was recorded in rheumatoid arthritis (RA) characterized by persistent and progressive diarthrodial joint inflammation and destruction [61]. According to the proposed model, blood pGSN decline is caused by the re-distribution of gelsolin into the inflamed synovial joint space for its actin-severing purpose. Alternative explanations include binding of pGSN to some plasma factor, decreased production, or proteolytic degradation of GSN [61]. Despite the promising report by Osborn et al., the utility of the pGSN level as biomarker of RA is still debatable, largely due to contradictory results showing that the potential for gelsolin as a biomarker of RA is strongly dependent on the method used for detection, especially in urine [62,63]. It was hypothesized that using the patients’ urine to evaluate the activity and progression of RA could be an innovative diagnostic approach due to the high stability of urine when compared to other bio-fluids, and the possibility of collecting it routinely and non-invasively at home by patients in large quantities. Proteomic analysis of RA patients’ urinary samples using capillary electrophoresis-mass spectrometry (CE-MS) demonstrated that in RA patients, peptide fragments identified as gelsolin-derived were detected in lower concentrations compared to samples collected from healthy volunteers [62]. Moreover, quantitative urinary proteome profiling using liquid chromatography-tandem mass spectrometry (LC-MS/MS), integrated with gene expression data from joint tissues and peripheral blood mononuclear cell, classified gelsolin as a potential urinary biomarker [128]. Further studies did not confirm the predictive role of gelsolin; although the urinary levels of GSN differed significantly from samples collected from control subjects (over 2-fold increase of concentration), no significant association with disease status was noted and GSN levels did not show a statistically significant association with radiographic progression of RA [63]. Hu et al. reported that despite significantly lower plasma gelsolin levels in patients with RA, its potential clinical application in RA diagnosis and disease activity evaluation is limited since no correlation between plasma gelsolin levels and RA disease activity score 28 (DAS28) was observed [129]. Relatively weak effects showing the prognostic value of pGSN in ankylosing spondylitis patients undergoing anti-TNF-α monoclonal antibody-infliximab therapy were also noted. GSN serum levels were lower than healthy controls (with significantly lower concentrations for men), but no association between specific clinical features of the disease, adipokines, or biomarkers of endothelial cell activation were seen [130]. The decrease of gelsolin in the blood has been also reported in multiple sclerosis (MS) patients [67].

#### 2.4.2. Uncertain Significance of Gelsolin as a Predictor in Autoimmune Diseases

The assessment of gelsolin concentrations was also performed in chronic, autoimmune diseases with high inflammatory component, as well as allergic diseases. Gelsolin was reported to be released by epithelial cells into the airways by an IL-4-induced mechanism, and concentrations of both IL-4 and extracellular gelsolin were enhanced in the bronchoalveolar lavage of patients with asthma, where GSN fluidizes airway surface liquid by breaking down filamentous actin [10]. Allergen-specific immunotherapy increased plasma gelsolin levels in pollen-induced allergic rhinitis patients, in accordance with reports about anti-oxidant and anti-apoptotic features of this protein [131]. Another study by Eke Gungor et al. demonstrated decreased levels of pGSN in children with atopic dermatitis; however, no significant differences were noted between atopy-positive and atopy-negative patients, suggesting that the role of pGSN in atopic dermatitis disease could be associated with prevention of Fas-induced keratinocyte apoptosis, rather than IgE-mediated type 1 hypersensitivity reactions [64]. The potential clinical application of plasma gelsolin in disease diagnosis and activity evaluation was also proposed in patients with systemic lupus erythematosus [129] and Henoch–Schoenlein purpura [132]. The results of these studies are relatively unclear, and more compelling evidence is required for proper recognition of plasma gelsolin as a biomarker for autoimmune and chronic inflammatory disorders.

### 2.5. Neurological and Neurodegenerative Diseases

#### 2.5.1. Neuroprotective Role of Gelsolin in Ischemia-Induced Brain Injuries

A number of studies suggest the neuroprotective role of gelsolin and potential of hypogelsolinemia as a therapeutic target in the development for therapeutic interventions in some neurological illnesses, including ischemic stroke [133], Alzheimer’s disease [134], multiple sclerosis [67], tick-borne encephalitis and Lyme neuroborreliosis [66], and subarachnoid hemorrhage [135]. Furukawa et al. and Endres et al., using gelsolin knock-out mice, demonstrated that gelsolin protects neurons against excitotoxic Ca^2+^ overload via modulation of calcium influx through N-methyl-D-aspartate (NMDA) receptors and voltage-dependent Ca^2+^ channels (VDCC) in hippocampal neurons. This gelsolin-mediated mechanism prevents brain injury after ischemia/reperfusion due to stabilization of intracellular calcium levels, regulation of inflammation, and depolymerization of actin released from damaged cells in response to glucose/oxygen deprivation [33,133]. High amounts of F-actin released upon tissue injury increase the viscosity of blood and disturb flow through the microvasculature, suggesting that the actin-severing properties of pGSN are crucial to limit inflammation and decrease blood clogging. Thus, pGSN concentration might be employed as an additional plasma predictor for first-year mortality from ischemic stroke [133]. Enhanced gelsolin expression is also an important mechanism by which histone deacetylase inhibitor trichostatin A (TSA) protects against ischemic brain injury, since (i) up-regulation of GSN was observed simultaneously with increased histone acetylation and (ii) TSA pre-treatment decreased levels of filamentous actin in brain of wildtype but not gelsolin-deficient mice [136]. Administration of exogenous gelsolin was also proposed to limit neuroinflammation and apoptotic signaling in mice following thermal injury [84].

#### 2.5.2. The Protective Role of Gelsolin in Alzheimer’s Disease and Its Potential as an Indicator of Rapidity of Cognitive Decline

A number of reports indicating additional beneficial properties of pGSN, including anti-amyloidogenic, anti-oxidant, and anti-apoptotic, are encouraging with respect to a role for gelsolin in diagnosis and prognosis of early-stage age-related Alzheimer’s disease (AD) [137,138]. Both cytosolic and plasma gelsolin bind amyloid beta protein (Aβ), a major component of amyloid plaques in the brains of individuals with AD, inhibit its fibrillization, and solubilize preformed fibrils of amyloid beta [139]. Data from various experimental models suggest that GSN intraperitoneal administration or peripheral transgene expression of pGSN reduce neuronal loss and amyloid load in the brain. Potential mechanisms include reducing oxidative stress, limiting Aβ fibrillogenesis both intra- and extracellularly, sequestering Aβ from the periphery, inhibiting Aβ-induced neurotoxicity, and acting as an anti-apoptotic factor [88,89,140,141]. Accordingly, the concentration of plasma gelsolin was significantly reduced and positively correlated with the rapidity of cognitive decline in clinically diagnosed AD patients [134,142]. The expression level of GSN in the brain of AD patients is controversial, and the results obtained with human brain studies are contradictory. In contrast to research by Güntert et al. reporting no significant changes in brain gelsolin levels between dementia patients and non-AD patients [142], Ji et al. demonstrated decreased gelsolin levels and increased 48 kDa carboxyl-terminal fragments (gelsolin-CTF) in the frontal cortex of individuals. The latter probably result from proteolytic cleavage of GSN by caspase-3 during apoptosis, correlated with the severity of AD [143]. Downregulation of GSN was also noted in the AD cerebellum [144]. Proteomics studies revealed diminished levels of GSN in CSF of mild declined AD patients, but this has not yet been validated by subsequent immunoassays [65]. Unfortunately, these alterations are not specific for AD, which limits the use of gelsolin as a single predictive protein for this illness [145]. Some reports, however, suggest the utility of a combined biomarker panel consisting of pGSN level, the total amount of Aβ42 and Aβ40, plasma MMP3 activity, and clinical data to obtain high sensitivity and specificity of AD diagnosis and prognosis [146].

#### 2.5.3. Pgsn-Mediated Limitation of Neuroinflammation as a Therapeutic Approach in Neurological Conditions

Changes in blood concentrations of gelsolin (without changes in CSF levels) can result from inflammatory reactions induced by central nervous system infections, such as tick-borne encephalitis (TBE) or Lyme neuroborreliosis (LNB), as presented by Kułakowska et al. [66]. Despite the fact that TBE and LNB are caused by viral and bacterial factors, respectively, in both of these cases, changes in pGSN levels were similar (~20–50% of healthy controls). This observation suggests that depletion of gelsolin is not pathogen-specific, but might rather result from non-specific inflammatory responses and actin release from axonal damage within the intrathecal compartment [66]. It was proposed that a similar mechanism is responsible for plasma gelsolin reduction in other neurological disorders with highly inflammatory components, such as multiple sclerosis (MS), in which actin-mediated depletion of gelsolin in blood (but not in CSF samples) was found and reported by Kułakowska et al. [67]. Considering that persistent neuroinflammation in the central nervous system (CNS) is accompanied with the pathological development of neurodegenerative diseases, including MS, limiting CNS inflammation might represent a prospective therapeutic tactic in neurological diseases [147]. More detailed research using a mouse model of multiple sclerosis, i.e., experimental autoimmune encephalomyelitis (EAE), confirmed a decrease of plasma gelsolin with subsequent increase of GSN in acute EAE brains. Possible mechanisms of the beneficial effect of treatment with exogenous pGSN include: (i) decline of extracellular actin, (ii) diminishing of inflammatory response, and (iii) lower oxidative stress due to limiting myeloperoxidase activity [148] in the EAE brain. Interestingly, recombinant human pGSN exerts its therapeutic effect not only during EAE induction but also on days 8 and 10, during development of neuroinflammation and onset of early symptoms. In addition, delayed treatment with rhp-GSN produced faster and more complete resolution of the clinical symptoms, which is likely determined by higher bioavailability of this protein [35].

#### 2.5.4. Proteolytic Cleavage of Gelsolin by Matrix Metalloproteinases in Subarachnoid Hemorrhage Patients

Along with the studies showing considerable prognostic value of gelsolin in predicting clinical outcomes in patients with acute brain injuries, including intracerebral hemorrhage [68], ischemic stroke [133], and traumatic brain injury [40], a number of studies demonstrate the usefulness of low levels of pGSN as an independent predictor of poor outcome after aneurysmal subarachnoid hemorrhage (SAH). In a study employing 262 SAH patients and 150 control subjects, Pan et al. revealed that pGSN is significantly reduced in SAH subjects and low levels of pGSN predict clinical severity of SAH with 80.8% sensitivity and 75.1% specificity [69]. In contrast to several previous reports cited above, it is strongly suggested that the decrease of GSN, particularly in the CSF compartment of SAH patients, is mediated by the proteolytic cleavage of gelsolin by matrix metalloproteinases (MMPs; particularly MMP-3, MMP-1, and MMP-9), the elevation of which is associated with a poor SAH outcome [149]. In accordance with this hypothesis, Chou et al. reported (i) novel degraded pGSN in CSF of SAH patients and (ii) inverse correlation between MMP-9 and pGSN levels in CSF, indicating a key role of disease-specific pGSN proteolytic cleavage in SAH pathophysiology [69,70].

### 2.6. Cancer

#### 2.6.1. Contradictory Effects and Expression of Gelsolin in Cancers

One of the most fundamental characteristics of malignant and transformed cells is the abnormal organization of the actin cytoskeleton. Consistently, gelsolin, as one of the best-known actin cytoskeleton-affecting agents, was found to be implicated in cancer development. However, due to a number of studies presenting opposing effects in tumor suppression versus oncogenesis, the correlation between gelsolin expression and tumorigenesis remains controversial. Indeed, contradictory results might have been observed even in the same types of cancer, possibly because the role of gelsolin differs during the course of tumor progression [150,151,152,153,154,155]. Although gelsolin was found to be downregulated in gastric [150], breast [156], colorectal [152,157], lung [158], prostate [159], kidney [160], ovarian [161], and pancreatic [154] cancers, there is also evidence that upregulation of gelsolin in tumors may promote aggressive behavior, and increased expression of this protein in certain tumors correlates with poor prognosis and therapy-resistance. The latter effect may be due to gelsolin’s modulation of several signaling pathways, including EGFR, PI3K, and Ras-PI3K-Rac, resulting in anti-apoptotic, proliferation-inducing, and pro-migratory function in some types of tumor cells [155,162,163,164,165]. Currently, the majority of cancer-related studies are focused on the role of cytoplasmic gelsolin; data about the prognostic value of extracellular gelsolin are considerably less abundant.

An intriguing study by Tsai et al. indicated the use of the secreted isoform of gelsolin as a biomarker of distant organ metastasis status in colorectal cancer (CRC) patients [71]. Proteomics-based analyses of plasma samples from patients upon diagnosis of their primary and metastatic tumors provided data about increased levels of pGSN in metastatic versus non-metastatic patients, which was additionally supported by the observation that colon cancer cells expressing and secreting more gelsolin into the extracellular medium are characterized by enhanced migration capabilities in vitro [71]. The exact mechanism of this pGSN-mediated effect was not determined, but the most promising theory assumes the interaction of secretory gelsolin with proteins in the extracellular environment. Such interactions may affect intracellular signaling and cell motility, which would be in agreement with previous reports indicating binding of GSN to fibronectin [28] and its interaction with β2 glycoprotein I and ɑ5β1 integrin [166].

The expression of gelsolin in ovarian cancer tissues and cell lines revealed considerably low levels of GSN, in contrast to its expression in the epithelium of normal ovaries and benign adenomas. Nevertheless, no significant association between gelsolin expression and other clinicopathological markers or patient survival was established, although it was suggested that GSN expression changes might be associated with tumor grade [161]. Investigation of the secreted proteome from high-grade serous ovarian carcinoma (HGSOC), benign ovarian lesion (BOL), and control samples confirmed earlier data showing less gelsolin secreted from HGSOC cells, compared with BOL and control cells. In vitro studies revealed that the transfection of gelsolin into HGSOC cells inhibited colony formation, suggesting a tumor-suppressive effect of gelsolin. Importantly, the serum expression level of gelsolin was significantly decreased in the HGSOC cohort of patients when compared to the control group, which could considerably improve the diagnostic possibilities in ovarian cancer patients [167]. Interestingly, a report by Lokamani et al. suggests that expression of gelsolin in tissues might differ from the serum proteomics results; in contrast to increased expression of gelsolin in the tissues of squamous cell cervix carcinoma, pGSN levels were found to be decreased in plasma samples from the same cohort of patients. However, the issue of distinguishing cytoplasmic and plasma isoforms of gelsolin still remains [168].

#### 2.6.2. The Usefulness of pGSN in Distinguishing Cancers from Chronic Diseases and as a Predictor of Therapy Outcome

Pan et al. using plasma samples from patients with pancreatic cancer, chronic pancreatitis, and healthy age-matched controls, presented that several biomarkers, including gelsolin, that were increased in the blood of pancreatic cancer cases compared to healthy controls. Gelsolin demonstrated an AUC (area under the curve) value greater than 0.75 in distinguishing pancreatic cancer from the controls. Importantly, serum gelsolin, particularly together with lumican, was proposed as a highly sensitive combined biomarker for distinguishing pancreatic cancer and pancreatitis (at 95% specificity gelsolin-lumican reached 80% sensitivity) [72]. Considering that chronic pancreatitis is the main reason of false positive results for pancreatic cancer detection, the development of a highly specific and sensitive biomarker for early detection seems to be particularly important [169,170].

Recently, it was suggested that analysis of the plasma proteome might lead to development of more specific diagnostic and prognostic standards in breast cancer patients. Scumaci et al. demonstrated reduced gelsolin in plasma samples of patients with hereditary breast cancer and correlated this observation with BRCA1 mutation status, suggesting that gelsolin expression is modulated by BRCA1-dependent recruiting of ATF-1 (i.e., factor binding to GSN promoter and negatively controlling its activity). Accordingly, association of plasma gelsolin levels with BRCA1 mutation status increased the sensitivity of this combined biomarker in early diagnosis of breast cancer [73]. Down-regulation of pGSN was also noted in breast cancer patients receiving their first chemotherapeutic cycle of doxorubicin. Considering the reports indicating pro-tumorigenic activity of gelsolin in breast cancers [171] and its implication in inflammation, its reduction in plasma samples suggests reduced invasiveness of cancer and might predict response to neoadjuvant chemotherapy [172].

#### 2.6.3. The Utility of Other pGSN-Containing Body Fluids in an Early Detection of Cancers

A few reports suggest the utility of gelsolin in body fluids other than plasma in early cancer detection. Ohnishi et al. demonstrated that changes in CSF composition, including extracellular gelsolin expression, are potential indicators of abnormal CNS states, such as astrocytomas. An over 2-fold decrease of CFS gelsolin concentration in glioblastoma (grade IV) was noted when compared to diffuse astrocytoma (grade II). These results were further confirmed by immunohistochemistry of fixed astrocytomas tissues, which indicated that gelsolin expression drops when the grade advances [74]. The observation of increased levels of a truncated gelsolin fragment in urine samples from papillary thyroid carcinoma (PTC) patients was also proposed as a non-invasive approach to discriminate patients with benign thyroid goiter (BTG) from those with PTC [173].

Alterations in plasma gelsolin concentrations were also detected in the case of diseases involving changes in sphingolipid contents in blood and bone marrow plasma such as acute myeloid leukemia (AML). Considering that (i) a number of glycosphingolipids (GSLs) is involved in the differentiation of AML cells [174] and (ii) GSN is one of the universal carriers/scavengers of S1P and ceramide (CER) binding protein [27,175], it was suggested that the average gelsolin concentration in the plasma of ALM patients differs from the levels detected in healthy subjects. Wątek et al. confirmed that gelsolin concentrations in blood and bone marrow samples from AML patients are significantly lower when compared to its concentration in plasma of healthy controls, which results from disturbed gelsolin-actin interaction, binding of gelsolin to cellular mediators, or modulation of gelsolin synthesis in response to actin release. Simultaneously, due to interactions of sphingolipids with gelsolin, it is suggested that significantly increased plasma levels of sphingolipids in patients with AML are closely associated with decreased plasma gelsolin; due to this fact, modifications of GSLs and GSN concentration profiles might be useful in the development of a new anti-AML treatment method [176].

### 2.7. Chronic Kidney Diseases

#### 2.7.1. The Impact of Decreased Levels of Gelsolin in Development of Kidney Diseases

Plasma gelsolin has also been implicated in the development and clinical outcome of patients with chronic kidney diseases (CKD). In a large study that included 150 chronic hemodialysis patients, pGSN levels were reduced to an average of 30–50% of normal values. Decreased gelsolin levels were correlated with progress of renal disease, 1-year mortality, and levels of circulating actin, detectable during analysis in those patients [75]. Particularly striking was that in patients with the lowest pGSN levels, there was a 9.8-fold increase in mortality compared to those with higher pGSN levels and undetectable circulating actin. There are likely several factors leading to decreased pGSN levels in hemodialysis patients. Since muscles are the major source of pGSN biosynthesis, and patients with end-stage renal disease (ESRD) often exhibit muscle depletion, malnutrition, hypoalbuminemia, and diffuse tissue injury, it is suggested that extracellular gelsolin declines due to both impaired synthesis and accelerated actin-related clearance of pGSN [75]. There are also some suggestions that plasma gelsolin might be implicated in dialysis-related protein-energy wasting (PEW) syndrome, and changes in pGSN might reflect improvement in the PEW state by nutritional supplementation or pharmacological therapy [177]. These reports indicate that plasma gelsolin might potentially be a useful clinical biomarker for patients starting hemodialysis and can be employed during considerations for renal transplantation [75,177]. In addition, a recent study demonstrated a potential association between gelsolin and aortic arch calcification (AAC). Blood levels of gelsolin were found to be lowered with the progress of AAC, which results in impaired resorption of calcification by osteoclast-like cells and imbalance between calcification inhibitors and activators [76], in agreement with previous reports implicating the role of gelsolin in bone homeostasis through interaction with osteopontin and the lack of bone resorption in gelsolin-deficient adult mice [178]. Repletion of gelsolin concentration might therefore lead to resorption of vascular calcification and be beneficial for hemodialysis patients.

#### 2.7.2. pGSN as a Pathological Factor in IgA Nephropathy

Several recent studies suggest a role for pGSN in pathogenesis of IgA nephropathy (IgAN), an immune complex glomerulonephritis and one of the leading causes of ESRD characterized by recurrent hematuria [167]. According to a study by Zhang et al., serum pGSN concentrations are lowered in IgAN patients and strongly negatively correlated with the progress and prognosis of IgAN, possibly due to gelsolin-mediated transforming growth factor β1 (TGFβ1) and oxidative stress regulation [168,169]. Further studies showed that in contrast to serum pGSN concentrations, glomerular levels of pGSN and the content of pGSN in renal tissue was significantly increased in IgAN patients. Considering data indicating that pGSN promotes the proliferation of MCs via an integrin α2β1 receptor-dependent mechanism and by facilitating cell mitosis in association with regulation of CDK2 and cyclin A expression, it was suggested that serum and glomerular pGSN levels might be considered as new markers for predicting IgAN progression and prognosis [169]. Moreover, the synergistic effect of pGSN and pIgA induced glomerular fibrosis via the TGF-β1/Smads signal transduction pathway was demonstrated recently [170], which highlights the need for assessment of both serum and tissue concentrations of gelsolin in IgAN patients.

### 2.8. Other Diseases

In addition to the well-documented effect of plasma gelsolin in trauma injuries, sepsis, infections, and neurological disorders, single studies present the potential of pGSN in other diseases, including diabetes [31], pre-eclampsia [171], congenital genetic syndromes [172], or rhabdomyolysis [173]. Despite the fact that the predictive value of extracellular gelsolin is not supported by a significant amount of research, its utility should be taken under consideration in further biomarker studies

#### 2.8.1. The Beneficial Role of Intracellular Gelsolin in Maintenance of Physiology of Pancreatic β-Cells: Anti-Diabetic Effect of Recombinant Gelsolin in an In Vivo Model of Diabetes

A number of studies indicate an association of the actin remodeling ability of intracellular gelsolin with function and survival of insulin-secreting β-cells [179,180,181]. According to Kalwat et al. and Tomas et al., GSN directly associates with the N-terminal portion of syntaxin 4 (Syn4; required for biphasic insulin secretion) and regulates insulin granule exocytosis, while gelsolin-mediated modulation of F-actin is required for glucose-dependent MAPK signal transduction involved in *β*-cell insulin secretion [179,181]. Further research confirmed the role of GSN in delaying apoptosis in pancreatic β-cells [180]. Consistent with reports indicating a beneficial role of intracellular gelsolin in the maintenance of pancreatic β-cell physiology, down-regulation of pGSN in serum samples of patients diagnosed with diabetes was found, as detected using liquid chromatography-mass spectrometry (LC-MS)–based proteomics analyses [77]. Recently, gelsolin was also detected as one of the proteins differentially expressed in diabetics with mild or moderate non-proliferative diabetic retinopathy (compared to diabetics without diabetic retinopathy), suggesting that plasma GSN might be employed as a biomarker for detecting early stage diabetic retinopathy [78].

Alterations in GSN levels are not useful for distinguishing type 1 diabetes from hyperglycemia caused by type 2 diabetes, as there are no significant differences between pGSN in samples from type 1 and type 2 diabetic subjects [77]. However, it has been proposed that subcutaneous administration of recombinant gelsolin might serve as a therapeutic approach in diabetes, since a positive correlation was noted between lower pGSN (~50% of normal values) and higher blood glucose levels and the percentage of glycated hemoglobin in both human and murine diabetic subjects [36]. Different truncated versions of rhu-GSN with the ability to depolymerize F-actin (e.g., GSN28-161 or GSN G1-G3, but not GSN56-161) reduced blood sugar levels with potency comparable to sitagliptin, a standard antidiabetic drug, clearly indicating that actin-depolymerizing properties are crucial for the antidiabetic effect of recombinant gelsolin. The authors suggest that depletion of available pGSN from plasma is the result of release of F-actin due to tissue damage and cell death [36].

#### 2.8.2. Changes of Gelsolin Concentration during Pregnancy

Some reports highlight the role of pGSN during pregnancy, in prenatal diagnosis and in pregnancy-related conditions, including pre-eclampsia (PE). Previously, gelsolin was established to be required for proper ductal morphogenesis in the mammary stroma, since female GSN-deficient mice have defects in mammary gland morphogenesis. The extracellular isoform of GSN was also found in mid-trimester amniotic fluid, where it serves as an anti-endotoxin protective factor, binding to bacterial LPS and inhibiting TNF-α-induced inflammatory responses [15]. Increased activity of the extracellular actin scavenger system is also noted during normal pregnancy [182]. Two pregnancy related hormones, human chorionic gonadotropin (hCG) and progesterone, significantly upregulate pGSN promoter activity in muscle cells of pregnant mice compared to the control animals, suggesting a role of this protein during pregnancy [183]. Consistently, lowered levels of pGSN were detected in the sera of pregnant women carrying fetuses with conotruncal heart defects at 14–18 gestational weeks [184]. Thorough validation might also confirm the usefulness of maternal plasma and amniotic fluid supernatants for the non-invasive prenatal diagnosis of Klinefelter syndrome [185], Down syndrome [186], or intra-uterine growth restriction [187].

#### 2.8.3. Impact of Reduced Levels of pGSN in Pre-Eclampsia Progression

The most advanced studies are focused on the establishment of pGSN’s role in pre-eclampsia, a hypertensive disease of pregnancy that complicates 3–5% of pregnancies, and is characterized by endothelial dysfunction, excessive platelet activation, and widespread multi-organ dysfunction [188]. Among a variety of haemodynamic, placental, and maternal factors implicated in pathophysiology of pre-eclampsia, including (i) high levels of anti-angiogenic factors, such as soluble fms-like tyrosine kinase-1 (sFlt1) of placental origin and (ii) systemic release of necrotic, apoptotic, or aponecrotic syncytiotrophoblastic material (STBM) from the placenta [189,190,191], it is becoming clear that the development of early- and late-onset pre-eclampsia is also strongly associated with the unbalanced toxic effects of free circulating actin, despite the increased extracellular actin scavenger system (EASS) activity during pregnancy [182]. Accordingly, pGSN levels fall during PE pregnancy, which is associated with clearance of actin complexed gelsolin and/or cleavage by matrix metalloproteases resulting in loss of its actin-binding capacity [80]. A contribution of decreased pGSN levels in PE progression due to inactivation of actin and inflammatory mediators was also confirmed by Nadkarni et al. [79]. This study also demonstrated a novel protective effect of pGSN in PE pregnancy by acting as a negative regulator of syncytiotrophoblast extracellular vesicle shedding in both placental explant cultures and dynamic mechanical stretch studies. Exogenous recombinant pGSN at 100 µg/mL and 500 µg/mL strongly reduced spontaneous STBM dissemination and sFlt1 release from normal and pre-eclamptic placental explants, respectively, indicating that pGSN repletion might be taken under the consideration in potential therapy of pregnant patients with pre-eclampsia [79].

#### 2.8.4. Elevated Levels of Gelsolin in Rhabdomyolysis and Gelsolin-Related Familial Amyloidosis of Finnish Type

In contrast to low gelsolin levels in the majority of pathological conditions studied thus far, increased levels of serum gelsolin were observed in patients suffering from rhabdomyolysis, where induced synthesis secondary to actin release and/or liberation of GSN from gelsolin-actin complexes explains the increased extracellular gelsolin [81]. Similarly, elevated levels of serum gelsolin have been found in gelsolin-related familial amyloidosis of Finnish type (FAF), caused by a single base mutation in plasma gelsolin, which results in impaired gelsolin-actin interactions [82].

#### 2.8.5. Gelsolin as a Universal Inflammatory Biomarker and Therapeutic Tool: Future Directions

The discovery, development, and validation of novel biomarkers that can predict clinical outcomes in health conditions is a significant challenge for modern medicine. Due to the heterogeneous nature of a variety of diseases, the difficulties in distinguishing them from other similarly manifested conditions, long symptom-free early phases in some chronic illnesses, and the need for rapid start of therapy, well-documented biomarkers have the potential to serve a crucial role by providing adjunctive information to guide clinicians to timely, accurate diagnosis. In this context, altered concentrations of the extracellular isoform of gelsolin, prevalent mainly in blood plasma, but present also in other body fluids, has been proposed. Differential levels of pGSN have been observed in a variety of conditions, ranging from trauma, infections, and infection-associated diseases, including sepsis to chronic inflammatory and autoimmune disorders. Due to the broad spectrum of conditions in which plasma gelsolin seems to be implicated, extracellular gelsolin should be considered as a universal predictor for general health capable of predicting clinical outcomes in a variety of health conditions, rather than a specific biomarker for a particular disease. Importantly, the recovery of pGSN levels might be employed to measure the effects of therapy, as presented in malaria patients treated with chloroquine [56], ankylosing spondylitis patients undergoing anti-TNF-α monoclonal antibody-infliximab therapy [130], burn patients [49], or after allergen-specific immunotherapy [131]. Similarly, as suggested by a compelling number of rodent models and clinical trials, systemic administration of exogenous gelsolin or application of GSN in the form of aerosol substantially improves the clinical outcomes and survival in CF patients [86,125]. Given the above, three major conclusions might be drawn: (1) in a majority of diseases, decline of plasma gelsolin precedes, and therefore might predict, tissue and organ injury, and can be a predictor of critical care complications; (2) alterations in pGSN concentrations are primarily associated with actin scavenging and anti-inflammatory features of gelsolin, but other unrelated mechanisms should also be taken into consideration; (3) hypogelsolinemia to ~25% of normal value is strongly correlated with exacerbation of disease severity and increased mortality; (4) repletion with exogenous recombinant gelsolin to normal gelsolin values seems to be a promising therapeutic approach in a number of health conditions; (5) kinetics of GSN recovery might reflect the efficiency of therapy; and (6) extensive validation of the utility of gelsolin as a combined biomarker, together with other proteins (e.g., lumican in pancreatitis and pancreatic cancer diagnosis) are justified in order to augment the sensitivity and specificity of gelsolin-based biomarkers.

Despite the promising results presented from collected research, a number of limitations and inaccuracies must be considered for appropriate validation of extracellular gelsolin levels as a predictor of clinical outcomes. First, it is crucial to determine whether a decline of gelsolin concentration is specific for a particular disease, and not only associated with other un-related factors, such as hypoalbuminemia or malnutrition, resulting in reduction of muscle mass, and thus a decrease of overall gelsolin synthesis. Wang et al. in their study on critically ill surgical patients confirmed that the decrease of plasma gelsolin level was significantly stronger than that of albumin, which indicates that pGSN-related reduction was specific for severe sepsis, and not a simple consequence of systemic plasma protein loss or dilution [17]. In contrast, in chronic hemodialysis patients, serum albumin independently correlates with pGSN levels [75]. There is also the possibility that intracellular gelsolin, which is non-specifically released from injured and damaged cells, interferes with measurements of the concentration and activity of plasma gelsolin. In this case, detected alterations in gelsolin concentration might reflect not a direct decrease or increase of pGSN, but changes in total systemic level of gelsolin. To address this issue, we suggest the simultaneous measurement of cell damage, in order to limit potential interference in results. At the same time, synchronized measurement of serum actin and GSN might give additional information on the severity of systemic SIRS and sepsis, as suggested by Horváth-Szalai et al. [102]. Alternative approach to resolve this issue might be pGSN-specific assays; however, the majority of commonly used antibodies and analytic techniques do not distinguish cytoplasmic and secretory gelsolin. Reports about high variability in the immunoreactivity of commercially available antibodies, and the variability in recognition of gelsolin originating from CSF or plasma depending on the specific antibody employed, are also disturbing [192]. Due to different functions of plasma and cytoplasmic gelsolins [16,20], differentiation of these isoforms is crucial for proper validation of clinical utility. The use of monoclonal antibodies recognizing exclusively the extracellular isoform of GSN, or primers that flank unique sequences of plasma gelsolin would be a good approach, although one that has only been sparsely employed [193,194].

A significant problem that questions the usefulness of extracellular gelsolin as a diagnostic indicator in some diseases are inconsistencies between the results published by various research teams. The connection between low pGSN levels and the development of ARDS remains uncertain; research performed by Lee et al. did not confirm results obtained earlier in patients after major trauma (Mounzer et al.) [18,46]. Different conclusions about gelsolin levels detected in brains of individuals with Alzheimer’s disease were also noted by Güntert et al. [142] and Ji et al. [143]. Similarly, Saraswat et al. noted lower differences between gelsolin levels in pancreatic cancer (PC) versus chronic pancreatitis (CP) patients than Pan et al. [195]. These discrepancies might be associated with (i) different statistical analysis models, (ii) inclusion of more heterogeneous patient populations with varied disease severity, and (iii) employment of different estimation methods. Particularly, evaluation of extracellular gelsolin values in cancer patients is problematic, since these levels may vary during the course of tumor progression. The role of secreted gelsolin in cancer is still poorly understood, and all statements on predictive value of pGSN in cancers should be treated with caution. It should also be noted that all analytical methods for estimation of pGSN concentration (i.e., ELISA immunoassay, western blot, nucleation assay, severing assay, and chromatography-based analysis) have their own advantages and drawbacks. In HIV-1-infected individuals with or without cognitive impairment, application of SELDI-TOF analysis followed by weak cation exchange chromatography and one-dimensional electrophoresis allowed for the detection of different expression of gelsolin, unrecorded previously in the same cohort of samples using 2-DE difference gel electrophoresis (2-D DIGE) analysis [60]. Notably, none of these methods are routinely applicable in clinical settings due to the high cost of reagents, equipment, significant personnel involvement, and the long duration of the analysis. Considering this, novel reports about the development of an automated serum gelsolin immune turbidimetric assay for possible clinical use are particularly interesting [104].

The most pressing problem seems to be the low reproducibility and high variations in assessment of pGSN levels in both healthy subjects and ill patients. pGSN levels observed between samples taken from healthy volunteers may vary up to 2–3-fold depending on the estimation method and the number of healthy individuals [18,61,75]. Importantly, significant variation is observed even if the same measurement method is used [55,90], underlining the need for determining the correct physiological baseline for pGSN in the healthy patient control group, also as a function of age, gender, race, etc.

Despite a large and growing body of work, there is still much that is not understood about the value of plasma gelsolin as a diagnostic factor and therapeutic tool. Many reports are based only on proteomics studies in rodent models of disease, and thus must be thoroughly examined in clinical settings. In addition, these studies should not focus only on individual gelsolin features, e.g., actin filaments shortening, but also on other putative beneficial gelsolin-mediated mechanisms. In particular, modulation of LPS, LTA, and host inflammatory mediators by physiological or pathological levels of extracellular gelsolin seems to be worthy of consideration. The question remains if gelsolin-derived peptides, including PBP10, might replace full-length gelsolin in therapeutic interventions. The rapid development of nanotechnology-based techniques provide also possibility to engage nanomaterials as carriers of GSN in order to increase LPS/LTA binding efficiency via modification of their solubility, retention time, and penetration through biological barriers. Pharmacokinetic studies and continuation of clinical trials would also provide data about the dosage of exogenous recombinant gelsolin and increase the knowledge about the potential of pGSN in substitution therapy.

## Figures and Tables

**Table 1:** Changes in plasma gelsolin concentration in selected diseases evaluated using human samples and suggested mechanisms of pGSN concentration changes.

	Disease	pGSN	Material	Detection Method	Suggested Mechanism of pGSN Changes	Ref.
Trauma	Major trauma	↓	blood	western blot	binding of actin from damaged cells, formation of actin-gelsolin complexes	[18]
	Critically ill patients	↓	blood	nucleation assay	binding of actin from damaged cells, formation of actin-gelsolin complexes	[46]
	Burns	↓	blood	ELISA	binding of actin from damaged cells, formation of actin-gelsolin complexes, binding of inflammatory mediators, proteolytic cleavage by MMPs (*)	[12,47,48]
	Traumatic brain injury	↓	blood	ELISA	actin binding, formation of actin-gelsolin complexes, binding of inflammatory mediators and diminishing of neuroinflammation	[50]
	CPB-ALI	↓	blood	ELISA	actin binding, formation of actin-gelsolin complexes, binding of inflammatory mediators	[51]
	Acute liver injury	↓	blood	ELISA	binding of actin released from injured liver	[53]
Infections and infectious-associated diseases	Sepsis	↓	blood	ELISA, nucleation assay	binding of actin from damaged cells, formation of actin-gelsolin complexes, binding of inflammatory mediators	[17,54,55]
	Malaria	↓	blood	nucleation assay, severing assay, western blot, LC/MS/MS	binding of actin released from destroyed erythrocytes, binding of hemozoin and formation of hemozoin-gelsolin complexes	[56,57]
	HBV-induced cirrhosis	↓	blood	2-DE, MS/MS	not defined	[58]
	HAD	↓	CSF	2-DE, 2-D DIGE, western blot	not defined	[59]
		↑	blood	SELDI-TOF	not defined	[60]
Chronic inflammatory diseases	Rheumatic arthritis	↓	blood, synovial fluid	nucleation assay	distribution of gelsolin into inflamed synovial joint space, binding of actin from damaged cells, formation of actin-gelsolin complexes, decreased production (*), proteolytic degradation (*), binding to plasma factors (*)	[61]
		↓	urine	CE-MS	not defined	[62]
		↑	urine	ELISA	not defined	[63]
	Atopic dermatitis	↓	blood	ELISA	binding of inflammatory mediators, prevention of Fas-induced keratinocyte apoptosis (*)	[64]
Neurological disorders	Alzheimer’s disease	↓	CSF	2-D DIGE, MS, ELISA	not defined	[65]
	TBE, LNB	↓	blood	western blot	binding of actin from damaged cells, formation of actin-gelsolin complexes, binding of inflammatory mediators	[66]
	Multiple sclerosis	↓	blood, CSF	western blot	binding of actin from damaged cells, binding of inflammatory mediators and diminishing of neuroinflammation	[67]
	SAH	↓	blood, CSF	ELISA, western blot	proteolytical cleavage by MMP-3, MMP-1 and MMP-9	[68,69,70]
Cancers	Colon cancer	↑	blood	ELISA, western blot	interaction with extracellular environment proteins, increase of colon cancer motility	[71]
	Pancreatic cancer and pancreatitis	↑	blood	SRM, ELISA	not defined	[72]
	Breast cancer	↓	blood	LC-MS/MS, western blot	BRCA1-dependent recruiting of ATF-1	[73]
	Astrocytoma	↓	CSF	2-DE, MALDI-TOF/TOFMS	cleavage by caspase activity	[74]
Other diseases	Chronic kidney disease	↓	blood	nucleation assay	impaired pGSN synthesis due to muscle wasting, actin-mediated increase of gelsolin clearance	[75]
	AAC	↓	blood	ELISA	not defined	[76]
	Diabetes	↓	blood	LC-MS	binding of actin from damaged cells, formation of actin-gelsolin complexes, protein anabolism	[77]
	Diabetic retinopathy	↓	blood	SQ-MRM, SID-MRM	not defined	[78]
	Pre-eclampsia	↓	blood	ELISA	binding of actin from damaged cells, formation of actin-gelsolin complexes, proteolytic cleavage by MMPs	[79,80]
	Rhabdomyolysis	↑	blood	radioimmunoassay	induced synthesis, liberation of GSN from gelsolin-actin complexes	[81]
	FAF	↑	blood	nucleation assay	impaired gelsolin-actin interactions resulting from mutation in pGSN	[82]

Abbreviations: ELISA: enzyme linked immunosorbent assay; CPB-ALI: acute lung injury (ALI) induced by cardiopulmonary bypass; LC/MS/MS: liquid chromatography-mass spectrometry; 2-DE: two-dimensional electrophoresis; MS/MS: mass spectrometry analysis; HAD: HIV1-associated dementia; CSF: cerebrospinal fluid; 2-D DIGE: 2-DE difference gel electrophoresis; SELDI-TOF: surface-enhanced laser desorption/ionization time-of-flight; TBE: tick-borne encephalitis; LNB: Lyme neuroborreliosis; SRM: selected reaction monitoring; SAH: subarachnoid hemorrhage; CE-MS: capillary electrophoresis-mass spectrometry; MALDI-TOF: matrix-assisted laser desorption ionization-time of flight; AAC: aortic arch calcification; SQ-MRM: semiquantitative multiple reaction monitoring; SID-MRM: stable-isotope dilution multiple reaction monitoring; FAF: familial amyloidosis of Finnish type; (*) potential mechanism, not confirmed during the course of the study.

**Table 2:** Effects of animal studies aiming to evaluate the therapeutic potential of exogenous recombinant gelsolin.

Disease	Used Animals	Mechanism of Therapeutic Action of Gelsolin	Ref.
Lung injuries	Mice, rats	decrease of acute inflammatory response, binding of inflammatory mediators, limitation of neutrophil migration, inhibition of neutrophil adhesion to endothelial surface, improvement of pulmonary microvascular functions	[38,83]
Burns and thermal injuries	Mice	decrease of acute neuroinflammatory response, binding of inflammatory mediators, decrease of elevated caspase-3 activity, improvement of peripheral T lymphocyte functions, regulation of oxidative response, shortening of bleeding time	[42,84]
Sepsis	Mice, rats	binding of free circulating actin released from damaged cells, decrease of acute inflammatory response, binding of inflammatory mediators	[85,86]
Pneumonia	Mice	decrease of acute inflammatory response, binding of inflammatory mediators, improvement of bacterial clearance by macrophages via NOS3-dependent mechanism	[87]
Alzheimer’s disease	Mice	decrease of apoptosis, regulation of oxidative response, limitation of Aβ fibrillogenesis and Aβ-induced neurotoxicity	[88,89]
MS/EAE	Mice	decrease of acute neuroinflammatory response, binding of inflammatory mediators, regulation of oxidative response	[35]
Diabetes	Mice	depolymerization of F-actin	[36]

*Abbreviations:* MS: multiple sclerosis; EAE: experimental autoimmune encephalomyelitis.
